# Microbiome profiles of non-responding and responding paired periodontitis sites within the same participants following non-surgical treatment

**DOI:** 10.1080/20002297.2022.2043595

**Published:** 2022-03-13

**Authors:** SJ Byrne, D Chang, GG Adams, CA Butler, EC Reynolds, IB Darby, SG Dashper

**Affiliations:** Centre for Oral Health Research, Melbourne Dental School, University of Melbourne, Parkville, Victoria, Australia

**Keywords:** Subgingival microbiome, periodontitis, *Treponema*, subgingival plaque, *Porphyromonas*

## Abstract

**Aim:**

Periodontitis is a site-specific, chronic disease treated by non-surgical debridement of subgingival plaque. We aimed to determine the microbiome of sites that did not respond to this treatment (NR) compared with paired good responding (GR) sites before and after treatment.

**Materials and methods:**

In a longitudinal cohort study, clinical parameters of disease and biological samples were taken prior to and 3 months after treatment. Twelve NR sites from six participants were paired with GR sites within the same participant. Subgingival plaque samples were subjected to bacterial community analysis using *16S rRNA* gene sequencing.

**Results:**

There were no significant differences in clinical parameters and microbial communities at baseline between GR and NR sites. Bacterial communities in deep pockets were dominated by a small number of species, notably *Porphyromonas gingivalis* and *Treponema denticola*. In NR sites three months after treatment there was no significant change in bacterial composition whilst there was a collapse in the abundance of pathobionts in GR sites.

**Conclusion:**

NR sites were not identifiable prior to treatment by clinical or microbiological parameters. Treatment failed to disrupt pathogenic bacterial community in NR sites. Targeted suppression of particular species should be considered to initiate community collapse and aid disease resolution.

## Introduction

Periodontitis is a bacterial-associated chronic inflammatory disease of the supporting tissues of the teeth and is the sixth-most prevalent health condition worldwide, affecting 10% of the population [1]. It is characterised by loss of periodontal bone and soft tissue support and can lead to tooth loss and impaired quality of life [2].

The tissue damage in periodontitis is the result of an uncontrolled inflammatory response to a dysbiotic microbial community [3]. Initial treatment via non-surgical debridement removes subgingival biofilm and calculus [4] with the goal being reduction of local inflammation and stability of the periodontal tissues [5]. In combination with excellent patient-maintained oral hygiene, non-surgical therapy can result in long-term periodontal stability [6–8]. However, depending upon the evaluation criteria used, up to 40% of sites may be non-responsive to treatment [9,10]. While clinical factors such as pocket depth, site location and tooth type, and patient factors such as oral hygiene and smoking can influence response to treatment [7,11,12], the subgingival microbial community may also influence treatment outcomes [13].

The periodontal pocket habitat supports diverse microbial communities [14]. While species including *Porphyromonas gingivalis* and *Treponema denticola* are consistently associated with clinical indicators of disease such as periodontal pocket depth and bleeding on probing [15–17] as well as predicting disease progression [18], comprehensive examination via *16S rRNA* gene sequencing demonstrates that microbial community profiles differ between states of health and disease [19–22].

Non-surgical periodontal treatment alters the bacterial community composition with a tendency to reduce the relative abundance of disease associated genera such as *Porphyromonas, Treponema* and *Tannerella* and increase in genera associated with periodontal health such as *Streptococcus, Rothia* and *Actinomyces* with a corresponding reduction in inflammation and periodontal pocket depth [13,23,24]. However, such findings are not consistent across all studies [25,26]. This may reflect the unfortunately common use of pooled plaque samples, and failure to distinguish between sites that respond differently to treatment.

Whilst the classification of periodontitis occurs at a patient level [27], periodontitis is recognized as a site-specific disease [28]. There is a paucity of studies evaluating the microbial communities in subgingival plaque in individual sites following periodontal therapy, and current studies are plagued with methodological issues limiting their findings. Pooling plaque samples or classifying response to treatment in participants rather than sites does not enable evaluation of microbial community profiles that may be associated with a good response, or a poor response to treatment at sites [29,30].

Therefore, the aim of this study was to compare the subgingival microbial communities before and after non-surgical debridement in both good response (GR) and non-responsive (NR) sites using a longitudinal cohort study with an intra-individual paired site design.

## Materials and methods

### Ethics approval

Ethics approval for this project was granted by the Human Research Ethics Committee of The University of Melbourne (Ethics ID: 1,750,598), Royal Dental Hospital of Melbourne (RDHM) (Dental Health Services Victoria, Ethics ID: 320) and Melbourne Dental Clinic (MDC) (The University of Melbourne). Informed consent was obtained from all participants who were aware they were free to leave the study at any time without compromising their future treatment. The study is registered with the Australia and New Zealand Clinical Trials Registry, registration number ACTRN12619001307190.

### Study participants

Participants were recruited from the periodontics department of the RDHM and MDC. To be eligible for the study, participants had been diagnosed with periodontitis as determined by at least two non-adjacent sites per quadrant with a pocket depth ≥ 5 mm [31]. Participants had not received comprehensive periodontal treatment prior to their referral to the clinic. Participants were excluded if they had a systemic condition affecting periodontal disease such as diabetes, used systemic antibiotics within the last 3 months, were pregnant or breast-feeding, had a condition that required pre-medication or use of non-steroidal anti-inflammatory drugs. All eligible participants were screened from their baseline periodontal chart recorded as part of their initial assessment at the periodontics department. Participants were exited from the study if they undertook a course of antibiotic therapy during the study, or if they wished to withdraw. Seventeen participants were initially enrolled in the study. One participant was exited from the study following prescription of systemic antibiotics, and another withdrew, leaving 15 who completed the study. The group consisted of eight females and seven males and their mean age was 50.5 ± 11.4 (range: 33–70).

### Periodontal examination and subgingival plaque sampling

At baseline and review appointments, full periodontal charts were recorded, consisting of full-mouth periodontal probing depths (PD), recession (REC), clinical attachment level (CAL), bleeding on probing (BOP) and mobility. All single-rooted teeth with PD ≥ 6 mm were identified from the baseline periodontal chart and designated for data collection. Plaque index (PI) [32] and modified gingival index (mGI) [33] were recorded for each site. Following clinical examination, supragingival plaque was carefully removed from each site and subgingival plaque collected using a single stroke with a sterile curette. Each plaque sample was immediately placed into 200 µL of sterile DNAse/RNAse free ultrapure water (Invitrogen, Grand Island, NY) on ice until transferal to −80°C for storage.

### Treatment protocol

All participants received oral hygiene instructions and non-surgical periodontal treatment in all quadrants. All participants were treated by a periodontist-in-training whereby all periodontal charts and non-surgical treatment were supervised by an experienced consultant periodontist. Each participant was treated by the same clinician at each visit. The review appointment was carried out 3 months after debridement of the last quadrant.

### Subgingival microbiome analysis

#### Site selection

Sites were selected for microbiome analysis based on their clinical response to non-surgical periodontal treatment. A non-responsive (NR) site was defined as one that at the review appointment exhibited no change or an increase in PD with BOP. A good response (GR) site was defined as one that exhibited a reduction in PD to ≤ 4 mm with an absence of BOP at the review appointment [34]. For each NR site, a matching GR site in the same participant was selected for analysis in this study. A total of 240 single-rooted sites exhibited pocket depths (PD) ≥ 6 mm at baseline, and 3 months after treatment 19 sites (7.9%) were assessed to be non-responsive (NR) (Supplementary Table 1). These sites were derived from 6 participants (3 females, 3 males); 2 of whom were smokers, and their mean age was 51.1 ± 8.7 years (range: 39–61). One NR site could not be paired up with a GR site within the same participant, leaving 18 matched pairs of plaque samples for microbiomic analyses. Three plaque samples did not yield adequate amounts of DNA for analysis and a further three matched pair sites produced inadequate DNA reads for at least one time point for the GR site and were excluded. Therefore, a total of 12 paired sites at two time points (48 plaque samples) from 6 individuals were analysed in this study.

#### 16S rRNA *gene sequencing*

Next-Generation Sequencing-based community profiling was used to analyze and quantify the microbial communities in subgingival plaque. This was performed at the Melbourne Dental School, using an Ion Torrent Personal Genome Machine (PGM™; Life Technologies) as previously described [35] except PCR amplification of the V4 region was performed for 30 cycles due to the low concentration of gDNA template. The datasets generated and analysed during the current study are available from the NCBI Sequence Read Archive (SRA) repository using BioProject accession number PRJNA786436.

#### Bioinformatics

Bacterial taxonomy was determined using the amplicon sequence variants (ASVs) clustering method, DADA2 (Divisive Amplicon Denoising Algorithm 2 [36];). In the DADA2 workflow, the *de novo* read counts for the ASVs were constructed through the incorporation of both the quality scores and sequence frequencies in a probabilistic noise model for nucleotide transitions on the Nephele web-based platform for microbiome data analysis [37]. ASVs of less than 75 bp in length and chimeras were filtered out. The remaining ASVs were then classified taxonomically using the Ribosomal Database Project (RDP) classifier, with the Human Oral Microbiome Database (HOMD) version 15.1 as the reference database. Finally, a BLASTN search against the eHOMD 16S rRNA RefSeq version 15.2 database [38] was carried out to assign a representative species to those ASVs without a species level annotation where the identity was ≥ 99%. During taxonomic identification, there were bacterial species that could not be differentiated by the V4 region analysis. These species are presented as slash calls, for example, *Actinomyces naeslundii*/ *johnsonii*/ HMT169/ HMT170/ HMT171/ HMT175, which will collectively be known as the *A. naeslundii* group. The *Fusobacterium nucleatum* group is comprised of *F. nucleatum*/ HMT203/ *naviforme. F. nucleatum* subspecies were not differentiated during this analysis.

#### Statistical analysis

Individual ASVs in each sample were used to both classify the microbial lineages present (grouped as species level taxa) and determine the relative abundances of each taxon, expressed as a percentage of the total number of bacteria present. Differences in the bacterial community over time were determined by comparing the changes in the relative abundances of taxa for matched NR and GR sites within a participant. Alpha (within-sample) diversity measures (Shannon, Fisher and Inverse Simpson) were estimated for each subgingival plaque sample. Descriptive statistics (mean, standard deviation and range) were calculated for all continuous variables and frequencies for all ordinal variables. Ordinal periodontal measurements were compared between NR and GR sites using the Stuart-Maxwell marginal homogeneity test. Alpha diversity measures were compared using linear mixed-effects models (LMM) and beta diversity measures were compared using the permutation manova method *adonis* in R. Stata (version 14.2), RStudio (version 1.4.1717) and R (version 4.1.2) were used for the statistical analysis.

## Results

### Baseline site-level clinical parameters

At baseline, the 12 matched NR and GR sites were similar when assessed by clinical measures. The mean PDs were 6.8 ± 0.7 mm and 6.8 ± 0.8 mm for NR and GR sites, respectively (LMM, p = 0.96). The difference in clinical attachment level (CAL) was 1.1 ± 1.7 mm in favour of GR sites (LMM, p = 0.08) (Table 1). Bleeding on probing was similar with all NR sites and 10 of the 12 GR sites bleeding. Mobility was slightly greater at NR sites (median = 2) compared to GR sites (median = 1) (marginal homogeneity test, p = 0.08). There was no difference between groups for PI, with both NR and GR sites having a median score of 2 (marginal homogeneity test, p = 0.8). For mGI, the medians for NR and GR were 3 and 2.5, respectively (marginal homogeneity test, p = 0.3).

**Table 1.** Clinical responses to treatment of NR and GR sites. Cell values are mean ± standard deviation (range)

	Sites	Pre-treatment	Post-treatment	Difference	Significance ^a^
Pocket Depth(mm)	NR(*n = *1*2*)	6.8 ± 0.7 (6.0–8.0)	7.3 ± 1.0 (6.0–9.0)	−0.6 ± 0.7 (−2.0–0.0)	*p = 0.13*
	GR(*n = *1*2*)	6.8 ± 0.8 (6.0–9.0)	3.3 ± 0.6 (2.0–4.0)	3.6 ± 0.9 (2.0–5.0)	*p < 0.0001*
	Difference	−0.1 ± 0.2 (−3.0–2.0)	4.1 ± 1.1 (2.0–6.0)		
	*Significance*^a^	*p = 0.96*	*p < 0.0001*		
Clinical Attachment Level (mm)	NR(n = 12)	8.8 ± 1.4 (6.0–11.0)	10.3 ± 1.4 (8.0–12.0)	−1.6 ± 1.6 (−6.0–0.0)	*p = 0.051*
	GR(n = 12)	7.7 ± 1.2 (6.0–10.0)	6.2 ± 1.3 (4.0–8.0)	1.5 ± 1.7 (−2.0–4.0)	*p = 0.008*
	Difference	1.1 ± 1.7 (−1.0–3.0)	4.2 ± 1.7 (2.0–7.0)		
	*Significance*^a^	*p = 0.076*	*p < 0.0001*		

^a^**S**ignificance levels obtained by linear contrasts after fitting a linear mixed-effects model (LMM) with response, time and response by time included in the model as fixed effects, and paired sites as a random effect.

### Site-level clinical outcomes

The NR sites exhibited a mean increase in PD of 0.6 ± 0.7 mm and a loss of clinical attachment of 1.6 ± 1.6 mm (LMM; p = 0.13 and p = 0.051, respectively). In contrast, the matched GR sites exhibited a significant improvement showing a mean PD reduction of 3.6 ± 0.9 mm and a clinical attachment gain of 1.5 ± 1.7 mm (LMM; p < 0.0001 and p = 0.008, respectively; Table 1). The PD and CAL measurements were also significantly different between NR and GR sites at post-treatment (LMM; p < 0.0001; Table 1).

### Microbiomic analyses

The subgingival microbiome of the 12 site pairs was characterised prior to, and 3 months after treatment. The 48 sequenced subgingival samples generated a total of 4.7 million *16S rRNA* V4 region gene sequences, an average of 97,676 sequences per sample (range: 16,273 to 528,637). A total of 1,306 ASVs were identified. Following the RDP classification and BLASTN searching against eHOMD, a total of 11 phyla, 85 families, 160 genera and 452 bacterial OTUs were identified, with 353 (78%) identified at the species level.

### Subgingival microbiome of deep pockets

**Figure 1.**
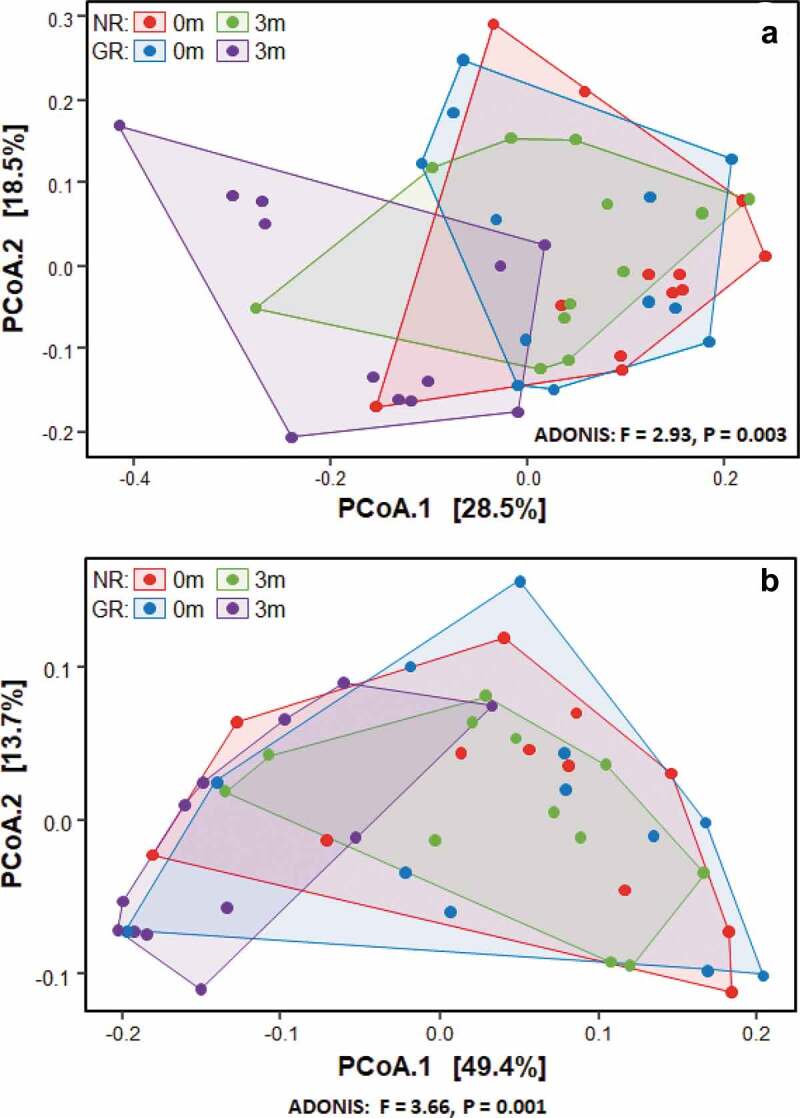
The effect of treatment on the bacterial composition of subgingival plaque as determined by Principal Coordinates Analysis (PCoA) using unweighted (**a**) and weighted (**b**) UniFrac distance measures. Samples were taken immediately prior to non-surgical periodontal treatment of matched NR (purple) and GR (blue) sites. Three months after treatment samples were again taken from the same matched NR (green) and GR (red) sites.

**Figure 2.**
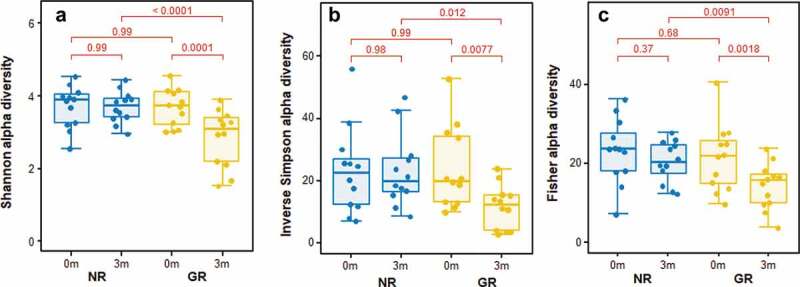
Comparison of subgingival plaque bacterial diversity before (0 m) and three months (3 m) after non-surgical periodontal treatment. All three analyses; Shannon (**a**), Inverse Simpson (**b**) and Fisher (**c**) showed a significant decrease in bacterial diversity after treatment in GR but not NR sites. There was no difference in bacterial diversity between GR and NR sites prior to treatment.

**Figure 3.**
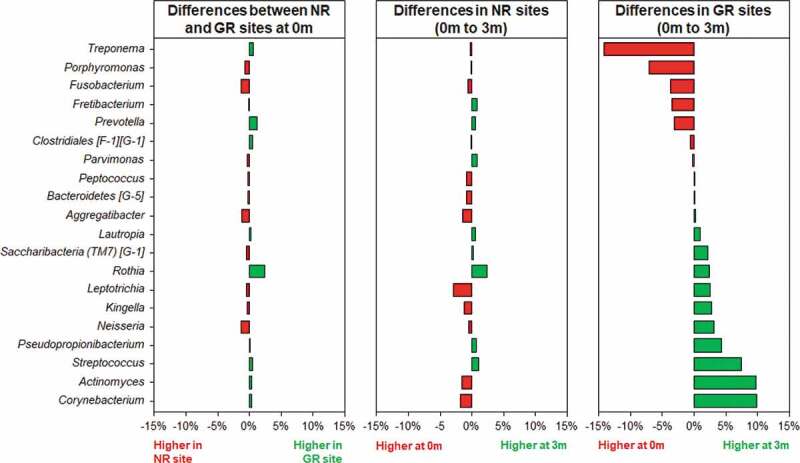
Differences in relative abundances of the 20 most abundant bacterial genera in subgingival plaque prior to and in response to non-surgical periodontal treatment in NR and matched GR sites. Subgingival plaque samples were taken immediately prior to treatment (0 m) and three months after treatment (3 m). There was little difference in relative abundance of any of the genera prior to treatment between the NR and matched GR sites. The x-axis depicts the relative change in abundance as a percentage of the total.

**Figure 4.**
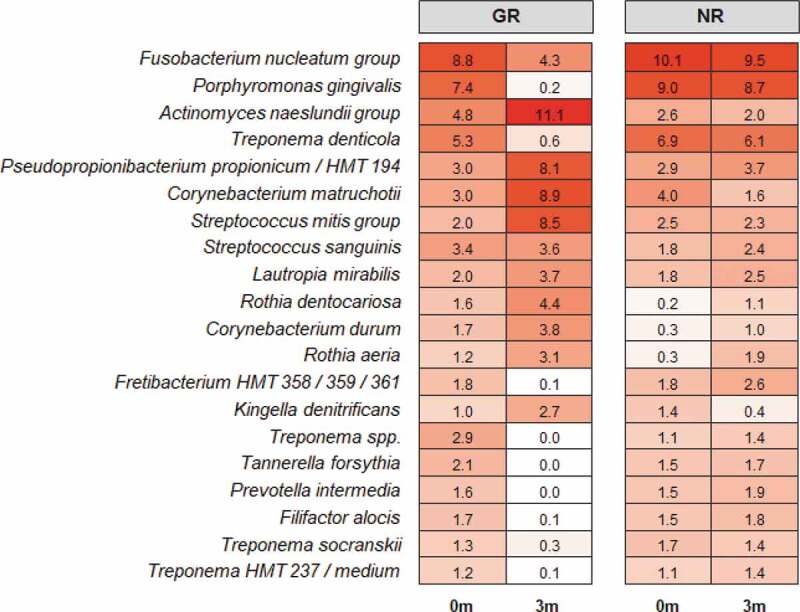
The relative abundances of the 20 most abundant taxa at a species level in subgingival plaque prior to non-surgical periodontal treatment (0 m) and three months after treatment (3 m) in NR and matched GR sites.

The beta diversity (differences between samples) was visualised in the ordination plots obtained from PCoA using both unweighted and weighted UniFrac distance measures (Figure 1). No significant difference was observed between the bacterial communities prior to treatment (pairwise permanova: unweighted – p = 0.78, weighted – p = 0.99). However, GR sites at 3 months had significantly different bacterial composition to the NR sites at 3 months, and all sites at baseline (pairwise permanova: unweighted – p = 0.003, weighted – p = 0.004). This was supported by the similarity of the α-diversity between these sites as determined using three separate indices, Shannon, Fisher and Simpson (Figure 2). Prior to treatment the most abundant genera in the subgingival microbiome at all sites were *Treponema* followed by *Fusobacterium* and *Porphyromonas* (Figure 3). Other abundant disease-associated genera included *Prevotella, Aggregatibacter, Tannerella* and *Filifactor*. At a species level the subgingival plaque community was dominated by a small number of taxa that accounted for >26% of all bacteria in these deep periodontal pockets prior to treatment. Both GR and NR sites were dominated by *P. gingivalis, T. denticola denticola*, the *F. nucleatum* group, and the *A. naeslundii* group (Figure 4).

### Microbiological changes after treatment

**Figure 5.**
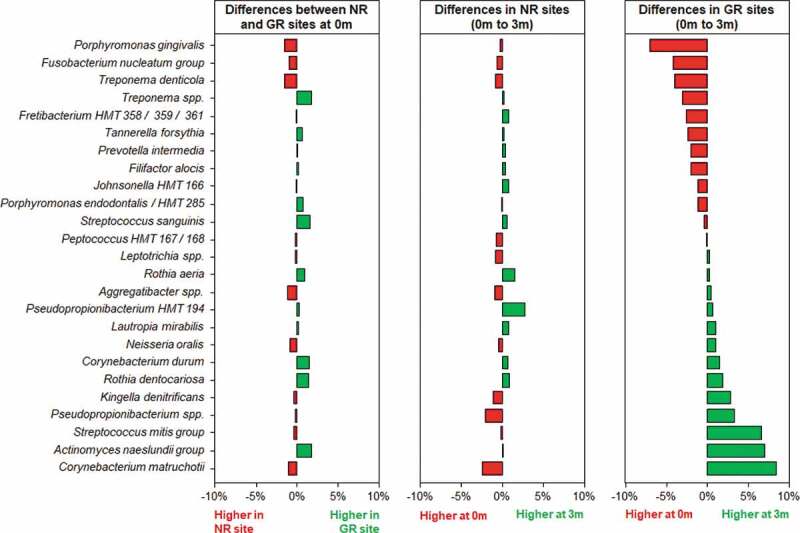
Differences in the relative abundances of the 25 most abundant bacterial taxa at a species level in subgingival plaque prior to and in response to non-surgical debridement treatment in NR and matched GR sites. Subgingival plaque samples were taken just prior to treatment (0 m) and three months after treatment (3 m). There was little difference in relative abundance of any of the taxa prior to treatment between the NR and matched GR sites. The x-axis depicts the relative change in abundance as a percentage of the total.

Three months after the non-surgical periodontal treatment, there was a significant decrease in bacterial α-diversity in GR sites as determined by all three measures of diversity (Figure 2) and a significant shift in the bacterial composition of the sites as determined by PCA (Figure 1). In contrast NR sites showed no significant decrease in bacterial diversity and the bacterial composition of the sites was comparable to pre-treatment. At a genus level, *Treponema* was the most abundant prior to treatment, however in GR sites three months after treatment they had decreased significantly (Figure 3). Similarly, there were notable decreases in the relative abundances of *Fusobacterium* and *Porphyromonas* and significant increases in the abundance of *Streptococcus, Actinomyces, Corynebacterium, Pseudopropionibacterium* and *Rothia* in these sites. At a species level the mean relative abundances of *Porphyromonas gingivalis, T. denticola, Tannerella forsythia, Fusobacterium alocis, Treponema socranskii, Treponema forsythia*, and *Fretibacterium* HMT were significantly reduced in GR sites (Figures 4, 5; Supplementary Figure). The *A. naeslundii* group, *Streptococcus mitis* group, *Corynebacterium matruchotii, Corynebacterium durum*, and *Rothia dentocariosa*, all increased significantly in sites that responded well to treatment. Interestingly *Rothia aeria* increased in abundance after treatment in both the NR and GR sites (Supplementary Figure). There were few significant changes in the abundance of individual species after treatment in the NR sites, although *C. matruchotii* significantly decreased in abundance and *Pseudopropionibacterium* HMT_194 increased significantly (Figure 4; Supplementary Figure).

## Discussion

Periodontitis is a site-specific disease, and its progression can be predicted by determining the composition of the subgingival plaque at that site, particularly the relative abundance of the pathobionts *P. gingivalis* and *T. denticola* [18]. In deep pockets, the subgingival microbiota is not homogenous, with *Treponema* species and *P. gingivalis* associated with the nutrient rich outer layer of subgingival plaque near the epithelial lining of the periodontal pocket [39,40]. Positioned adjacent to the inflammatory response being mounted by the host, these bacteria must be able to shield themselves from this response and are likely to do so by forming synergistic communities [41,42].

Management of periodontitis includes mechanical debridement, the aim of which is to remove bacteria from the subgingival environment. This therapy is generally effective at reducing inflammation, probing pocket depth and number of diseased sites in periodontitis patients [43]. However, these changes are not observed in all sites in an individual [30]. The ability to predict which sites will not respond to treatment and how sites do respond to treatment would be of value.

In this study, we found that even with meticulous debridement nearly 8% of the sites ≥ 6 mm in depth failed to respond to treatment; albeit a more favourable response than that has been reported previously [9,44]. The better than expected response to treatment in this study might be explained by the site selection. As response to treatment may be influenced by factors such as not only pocket depth but tooth type and furcation involvement. In this study the environmental variation in sites was reduced by including only single-rooted teeth with pockets ≥ 6 mm. Clinical parameters of disease at these sites prior to treatment were not able to predict which sites would respond poorly.

Most microbiological studies of response to treatment have provided limited information due to the pooling of subgingival plaque samples [23,45]. We overcame these limitations using a longitudinal study with a matched site approach using paired non-responsive (NR) and good response (GR) sites in each participant. Prior to treatment there were no significant differences in the microbial communities found at the GR and NR sites. This indicates it is currently not possible to predict which sites are likely to respond well or poorly to treatment based on their overall bacterial community composition immediately prior to treatment. Although not reaching statistical significance, the relative abundance of some taxa including *R. dentocariosa, R. aeria* and *C. durum* was lower in non-responding sites prior to non-surgical debridement. Both *R. dentocariosa* and *R. aeria* tend to be associated with health [46]. Further research is required to determine if threshold levels of these species support the development of subgingival microbial communities associated with disease resolution following periodontal therapy. The dominance of the genera *Treponema, Porphyromonas, Prevotella* and *Tannerella* in subgingival plaque communities prior to treatment, and *Streptococcus* and *Actinomyces* post-treatment in sites responding well to treatment is in agreement with previous observations [24].

In the sites that responded well to treatment a collapse of bacterial diversity (Figure 1, Figure 2) and a corresponding change in the relative abundance of disease and health associated microorganisms at both a genus and species level was observed (Figures 3, 4). This is likely to reflect the successful removal of the pathobionts residing in the deeper layers of the pocket, leading to the local resolution of inflammation and the subsequent change in nutrient profile which will enable the re-establishment of health-associated species and lead to a decrease in pocket depth and resolution of BOP. In GR sites, there was a decrease of 3.5 ± 0.8 mm in pocket depth, three months after treatment and resolution of BOP. These factors will further disadvantage the re-establishment of the known pathobionts *P. gingivalis, T. denticola, T. forsythia, Filifactor alocis* and *T. socranskii*. Prior to treatment these species represented a median of ~ 20% of all bacteria present in the plaque sample (Figure 5), and it is very likely that they comprised an even greater proportion of the microbiota in the deeper layers of the pocket; an environment that would preference their proliferation [40,47]. Treponemes dominated the subgingival microbiota prior to treatment but were significantly diminished in sites that responded to treatment (Figure 3). This reduction in diversity following debridement is not consistently observed in previous studies [24]. The use of pooled plaque samples in this type of study may hinder the observation of this reduction in diversity following treatment [48]. As the microbial diversity of subgingival plaque samples from healthy sites has been reported to be lower than that from diseased sites, the reduction in diversity upon successful treatment is consistent with that site being restored to health [49].

Although the microbial communities in both the NR and the GR sites changed following debridement, the change in GR sites was more pronounced (Figures 3, 4, and 5). The significant reduction in pathobionts and their associated virulence factors enabled clinical resolution of inflammation at a site. The microbial community three months post-debridement in these sites was dominated by *C. matruchotii, Actinomyces* species and the *S. mitis/oralis* group (Figures 3 and 4), species from genera that dominate healthy plaque [50]. In the NR sites, some disruption of the microbial community occurred (Figure 5) however not enough to sufficiently remove the inflammatory burden, resolve inflammation and prevent a re-emergence of pathobionts such as *P. gingivalis* and *T. denticola* in a diverse community (Figure 2). It is likely that the non-resolving inflammation post-treatment continues to provide an environment and nutrients that reinforce a dysbiotic microbial community, as exhibited by baseline levels of both disease and health associated species 3-months post-treatment.

In this study, we focused on the relative abundance of bacterial species in subgingival plaque. By the very nature of a periodontal pocket, the subgingival plaque biomass is greater in periodontitis sites than in health [49], with a corresponding increase in microbial challenge to the host. However, as discussed in a recent review of the role of the microbiota in periodontitis [46], evidence points to specific alterations in microbial community composition rather than the total microbial load being associated with periodontitis [22,51]. Despite the small sample size of the current study, these findings are in agreement with previous research where sites responding well to treatment exhibit a reduction in species diversity post-treatment [29], and a reduction in the relative abundance in the disease-associated genera *Porphyromonas* and *Treponema* [23,29]. The current study adds to the knowledge regarding microbial communities associated with sites that do not respond to treatment, demonstrating that these changes are not observed in non-responding sites.

## Conclusion

In this study, successful treatment was associated with a reduction in bacterial community diversity, and a significant reduction of periodontal pathobionts at 3-months post-treatment. Removal of these bacteria and their inflammation-associated virulence factors enables clinical resolution of inflammation at a site suggesting that targeted reduction of these species is necessary for resolution of inflammation, and to reduce the number of non-responding sites following periodontal therapy.

## Supplementary Material

Supplemental Material
