# Supplementary material for: Microbiome profiles of non-responding and responding paired periodontitis sites within the same participants following non-surgical treatment
Source: J Oral Microbiol. 2022 Mar 13;14(1):2043595. doi: 10.1080/20002297.2022.2043595 (PMC8920355; doi:10.1080/20002297.2022.2043595)
Supplement: Supplemental Material [file ZJOM_A_2043595_SM2866.docx]

**Supplementary Table 1.** Patient-level clinical parameters. PD = pocket depth, CAL = clinical attachment level and BOP = bleeding on probing.

|  | **Parameter** | **Pre-treatment** | **Post-treatment** | **Difference** |
| --- | --- | --- | --- | --- |
| All participants (15) | PD | 4.0 ± 0.6 mm  (3.0 – 5.4) | 3.1 ± 0.4 mm*  (2.3 – 3.9) | 1.0 ± 0.5 mm  (0.5 – 1.9) |
|  | CAL | 4.9 ± 0.7 mm  (4.0 – 6.1) | 4.7 ± 0.5 mm  (3.7 – 6.0) | 0.3 ± 0.4 mm  (-0.2 – 1.1) |
|  | BOP | 71.3 ± 18.0%  (38.0 – 94.7) | 35.6 ± 16.0%*  (11.8 – 66.0) | 35.7 ± 15.3%  (15.1 – 68.2) |
| Sequenced participants (6) | PD | 4.2 ± 0.6 mm  (3.4 – 5.4) | 3.4 ± 0.3 mm*  (2.9 – 3.9) | 0.9 ± 0.5 mm  (0.5 – 1.9) |
|  | CAL | 5.3 ± 0.6 mm  (4.3 – 6.1) | 5.0 ± 0.5 mm  (4.5 – 6.0) | 0.3 ± 0.4 mm  (-1.6 – 0.7) |
|  | BOP | 74.0 ± 13.8%  (51.5 – 94.7) | 36.6 ± 9.7%*  (18.9 – 48.6) | 37.4 ± 8.0%  (24.7 – 48.1) |

* Significantly different (p < 0.05) to pre-treatment value as assessed by unpaired t-test.

**Supplementary Figure.** The relative abundance of the 25 most abundant bacterial taxa at a species level in subgingival plaque prior to and in response to non-surgical debridement treatment in NR and matched GR sites. Subgingival plaque samples were taken just prior to treatment (0m) and three months after treatment (3m).
